# Addressing Adversities in Caring for Mental Health in Rural Settings: A Conversation with Rural Mental Healthcare Practice Co-founder Dr. Taryn S. Van Gilder-Pierce

**DOI:** 10.5195/ijms.2022.1636

**Published:** 2022-12-03

**Authors:** Ahmed Nahian, Jewel Shepherd, Taryn S. Van Gilder-Pierce

**Affiliations:** 1 Center for Brain and Behavior Research, University of South Dakota, Vermillion, SD, USA.; 2 University of South Dakota, Vermillion, SD, USA.; 3 Psychological Associates of Yankton, LLC, Yankton, SD, USA.

**Keywords:** Rural, Mental, Psychology, Telemedicine, Telerehabilitation (Source: MeSH-NLM)

## Abstract

Due to lack of mental healthcare facilities in rural areas, the population often resorts to private practice practitioners to address their need for mental health services. Dr. Taryn S. Van Gilder-Pierce and her husband, Dr. William D. Pierce founded their private practice in Yankton, SD in 2001. She has more than 25 years of training and experience treating individuals, married couples, families, and groups in rural South Dakota. The interview delves into the challenges faced by early career professionals in building a practice in remote areas and extends into the room for expansion within the field of rural mental health provision of services.

## Introduction

**Figure 1.**
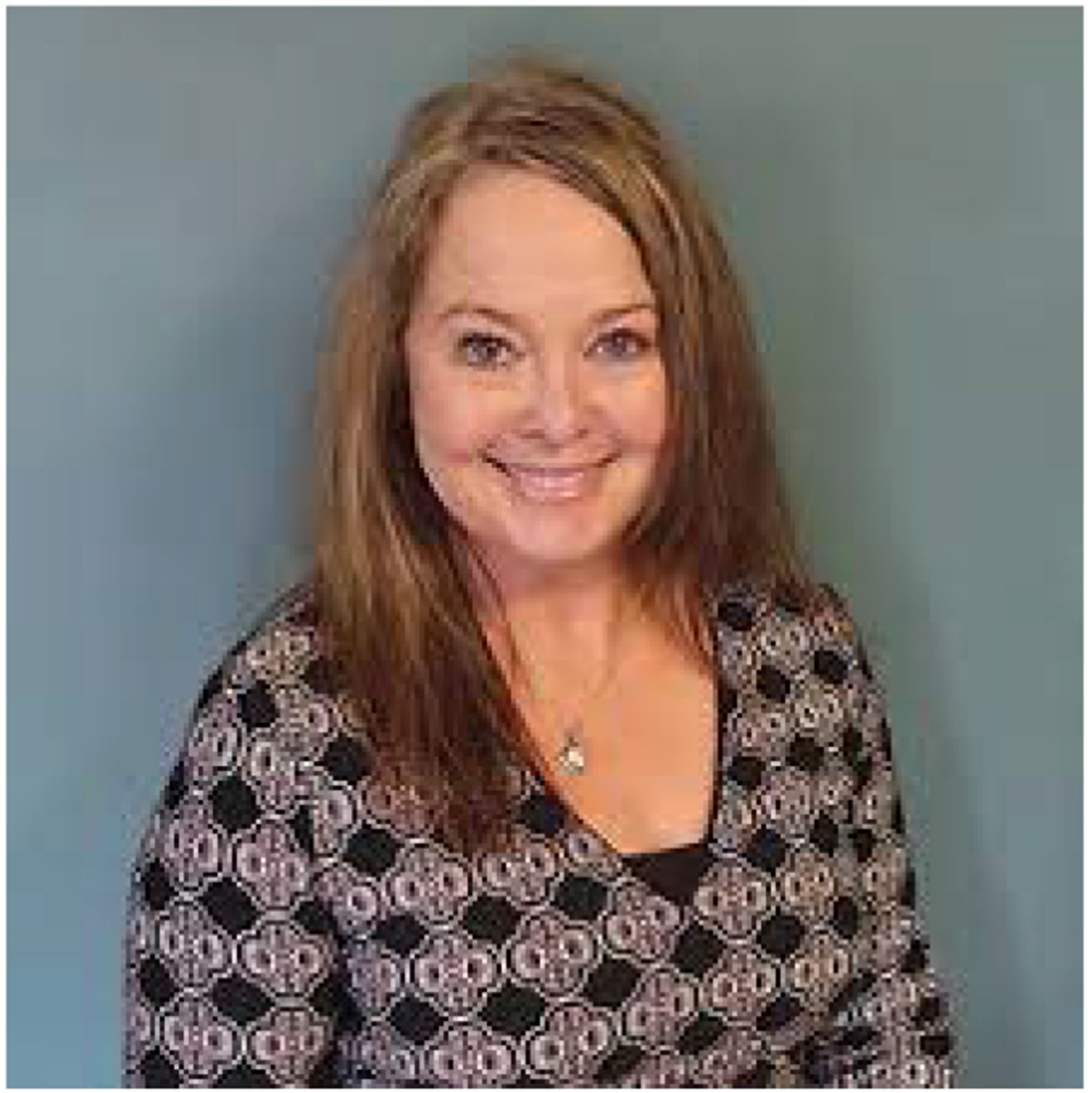
Dr. Taryn S. Van Gilder-Pierce’s professional picture provided by her practice.

The disparities between prevalence of mental health needs and access to healthcare in the rural and urban areas of South Dakota is concretely visible. ^1, 2^ The rural areas are 1.6 times more likely to be impoverished than the urban areas of the state. ^3^ With only 24 publicly funded outpatient community mental health center clinics across the state, the rural population often resorts to private practice professionals for treatment. ^4^ Taryn S. Van Gilder-Pierce, Ph.D. received her education in San Diego, California, and currently maintains a group private practice in Yankton, South Dakota (Figure 1). She co-founded the practice in Yankton in 2001 with her husband, William D. Pierce, Ph.D. Her 2.5 decades of experience offers a wide spectrum of expertise with competence in individual, couples, family, and group settings that provide a concrete picture of mental healthcare as it plays out in rural underserved areas. Diagnostic assessment and psychological evaluations are provided as diagnostic services facilitate therapy planning. Forensic services offered include child custody evaluations and various criminally related evaluations. Her group serves people of all ages, from infants to the elderly, and offers therapeutic intervention that is direct, active, and collaborative to maximize long-lasting effects.

### Ahmed Nahian (AN):

**Figure 2.**
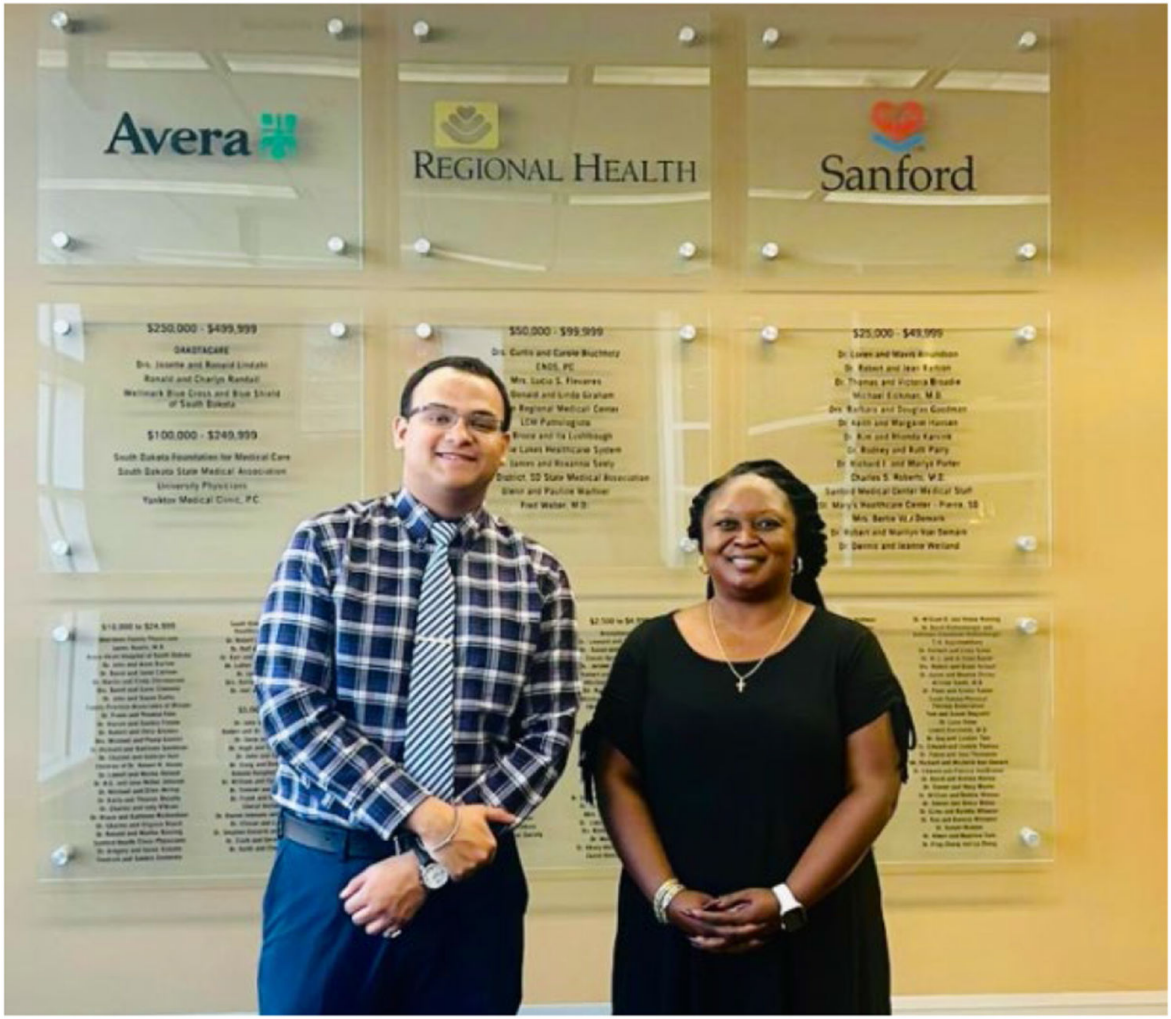
Ahmed Nahian, Recipient of the Summer Program for Undergraduate Research in Addiction (SPURA) Grant at the University of South Dakota, with His Mentor, Dr. Jewel Shepherd, After the Completion of His Summer Project Presentation in Lee Medical Building, Sanford School of Medicine, Vermillion, SD.

Good afternoon, Dr. Van Gilder-Pierce. I really appreciate you taking the time to meet with me. As part of the Summer Program for Undergraduate Research in Addiction (SPURA) here at the University of South Dakota, our team looked at the role of rurality and rural settings on substance use disorders (Figure 2). However, I am interested in mapping out caring for mental health in rural settings, in general. To start, can you tell us a bit about your career till now, your journey into caring for mental health, and why South Dakota as a practice location?

### Dr. Taryn S. Van Gilder-Pierce (TSVGP):

My husband and I were educated in California and did predoctoral internships in Little Rock and Cabot, Arkansas, respectively from where we were recruited into a small group practice headed by a psychiatrist in southern Arkansas. We spent about five years in that non-metropolitan setting with a practice radius of about 90 miles, which is common for practitioners in rural areas. While servicing a dynamic cohort of patients, I became more expansive in my practice serving most needs of individuals ranging from the child and adolescent years to geriatrics as well as custody evaluations, while my husband expanded his expertise in substance abuse and prison practice. When my husband received a psychologist position in Yankton with the federal prison system, we were able to start a private practice in an area of need with the security his job offered. Despite knowing no one, the practice grew rapidly, given the great need for services.

### AN:

What do you feel differentiates practicing in a rural setting as opposed to a setting in a metropolitan area?

### TSVGP:

You become a jack-of-all-trades when you practice in a rural setting. People want to be served locally. It is inconvenient for individuals seeking treatment or testing to drive to cities. I learned how to treat across the lifespan by practicing locally and expanding my areas of practice. Compared to my colleagues in the cities, who tend to be more specialized on their focus of care, I learned how to be more general while maintaining competency.

### AN:

What are the biggest challenges and rewards of practicing in a rural setting for you so far?

### TSVGP:

There would be different versions of experience depending on your role or mode of employment. I am an employer who also is an employee. As a self-employed private practice rural practitioner, I get to choose the array of services and who I serve while running a business. In my experience, rural providers get to see the benefit of their work in rural communities. People value your help and accessibility as there are so few practitioners to meet the tremendous amount of need. We, however, are on our own, which can be a disadvantage, especially in expanding to meet ever rising needs. Government grants are virtually impossible to acquire for private groups like ours, and we must self-fund a recruitment budget. We have less mobility for expanding our service provider team and overhead needs.

### AN:

In your perspective, what are the major features of a successful mental health caregiver in a rural setting?

### TSVGP:

I think one of the biggest factors would be resiliency. According to current research, practicing as a self-employed practitioner opens you up to more room for burnout. ^5^ Those of us who have been in this format of practice for a long time have been able to do so mainly for our resiliency; instead of feeling the need to work, you have the desire to work. Being truly engaged in the work feeds you rather than depletes you. It is not that the work is without stress but being energized by my work helps to preserve my personal mental health and secure my longevity of practice.

### AN:

What is the future of mental health in rural care? Do you think the government will take the initiative to provide opportunities for rural populations to have mental health support?

### TSVGP:

The government is trying, especially in South Dakota. The state has provided and is expanding mental health centers. In my experience, however, there are a lot of people who do not want a community setting and many prefer a private setting. The big push is to provide more private services offered by psychiatrists and psychologists by supporting and attracting them to rural areas. For example, in my field, the pre-doctoral internship offers a springboard into your career. It offers exposure to the location in which that internship is located and from where you may receive job leads. Although many states have an abundance of APA accredited pre-doctoral internships, South Dakota has only two internship placements at VA hospitals. In my understanding, pre-doctoral students who attend VA placements intend to remain working in the VA system. However, given that the VA system only serves veterans, attracting pre-doctoral students to South Dakota through the VA does not serve the nonveteran population. APA accredited internships are time-consuming for those institutions that offer them. Therefore, unless a large institutions with resources, such as a private hospital, begin offering pre-doctoral accredited internships, we must seek alternate avenues to recruit psychologists as well as psychiatrists to our state.

We were lucky enough to recruit an interested candidate, Alexandra Pagel, PsyD, to our team due to her desire to be closer to her family in North Dakota and Minnesota. Unless funding for training opportunities is provided in the private industry, we will not have an abundance of psychologists and psychiatrists interested in moving to rural communities.

### AN:

Can you tell us about the history of your practice and what led to its longevity?

### TSVGP:

We started in 2001 without any connection in the local area. As we planned to start the practice in January 2002, we listed our telephone landline at home under our business heading. We began getting calls for services shortly after listing it in summer 2001 and started the practice in November 2001 in advance of our previously planned opening. Starting from the scratch has its own benefits in that we could develop the practice in a manner that we felt best served community needs. The word-of-mouth strategy played a key role as did building strong referral relationships with area medical providers, other mental health providers, and attorneys. People want to be served locally and when you demonstrate competence, word spreads quickly.

### AN:

Do you think rural populations do not want to receive or do not have the means to reach out for mental health support?

### TSVGP:

We often talk about stigma in mental health, which is something that stands in the way of individuals seeking care. But, we forget to see the arial view of seeking mental health support. As a group of private practice psychologists, we do not see a large segment of indigent cases that are typically serviced for free or a greatly reduced rate through the local community mental health centers. Most people we serve therapeutically have private insurance, Medicaid, or Medicare. Legally related services are a cash product. For individuals who do not have some type of insurance, cash services are prohibitive, resulting in long waitlists at the community mental health centers. People who want services, sometimes, cannot get them due to a limited number of mental health professionals, in addition to a lack of financial resources. South Dakota developed a program to meet the needs of uninsured individuals in the state recently. However, we were willing to provide services through the program, because by history, individuals with no insurance and a lack of finances often sought services at our local community mental health center, and we had very few individuals seeking services through the program with our group. Although we were serving those who presented through the voucher system, we were discontinued as providers because our numbers were not high enough. The program was there, and we were willing to participate to meet needs of those without insurance population, but that opportunity ended for us.

### AN:

We recently underwent a global pandemic, and statistics indicate that more people reached out for mental health support. Can you tell us a bit about how the pandemic changed how you delivered mental health support to a rural population?

### TSVGP:

I have been practicing since the 90s, so the pandemic was a big learning experience for me as a practitioner. I had not previously utilized telehealth platforms to provide services. However, Dr. Pagel, who came from and trained at a metropolitan area, had experience with telehealth. Seeing what we were soon to face and having an experienced telehealth platform provider, we quickly made a smooth transition to a telehealth practice using Doxy.me, where you and I are meeting today. Patients were one click away from accessing care for their mental health, which is so fascinating now that I have experienced it. As the pandemic began subsiding, however, most people returned to in-person meetings.

### AN:

Our research found that adolescents living in rural areas are more likely to fall into substance-use disorders. Do you see this trend coming up in your practice?

### TSVGP:

Prior to my husband actively joining the practice following his retirement from the Department of Justice, we saw few treatment requests specifically for individuals with substance use disorders. Most of those needs are met through the community mental health center that has an extensive drug and alcohol treatment program and serves legally required substance evaluations. However, since establishing a caseload, my husband’s expertise in substance abuse is being sought with a large segment being adolescents. In our group, we are seeing referrals for youth resorting to chronic marijuana use.

### AN:

While psychiatry and psychology are clinically proven and revered in the medical field, there is still a stigma in seeking care for mental health. What is the current state of people’s trust in mental healthcare professionals?

### TSVGP:

When someone reaches out for treatment, despite the stigma that remains a clear part of the rural community, the biggest hurdle is trust. People who fear being ridiculed for their mental health conditions want utmost privacy. Although, in the mental health field confidentiality is a given, being in a setting that does not suggest exposure by being seen by people they may know is sometimes hard to navigate in a small town. Some individuals are not as affected by stigma and own their mental health whereas others are more challenged to seek help. Privacy beyond confidentially is vital.

### AN:

Why should aspiring psychiatrists and psychologists consider practicing in rural settings?

### TSVGP:

As a career, rural healthcare can be fruitful. In the private sector, compensation is competitive. The setting is very peaceful. Rural settings are also engaging because you can become actively involved in the locality. It is a meaningful life.

### AN:

What are ways interested students can get involved with mental healthcare professionals in rural settings?

### TSVGP:

Students can reach out to volunteer most times with any practice setting. We have seen a rising demand for paid experiences, however. For those needs, I would suggest that students look to their university programs to contract with local entities. In the past, we have employed Ph.D. students as testing technicians to help with our heavy testing caseload. Although not a full training experience, it gave them hours needed in test administration. With that connection, we recently have been designated as a testing practicum training site for the University of South Dakota. With insurance-based testing services, we can bill for a certain amount of the student’s time and thus, offer a paid position with the supervision required for a full training experience, something we did not have the luxury to do in the past. Also, as another example, you have funding from SPURA to conduct this project.

Living with unfulfilled or inadequately treated mental health needs has detrimental repercussions that are disproportionately felt by rural Americans. This long-standing issue's characteristics are clearly described by the need for care highlighted in Dr. Van Gilder-Pierce’s story. Starting a practice from the scratch brought along many obstacles that initially challenged the flow of her practice, but she saw that areas of need should have the necessary help they deserve. The anticipated effects on rural mental health will be achieved through research that examines novel treatment systems for rural populations, evaluates suicide prevention techniques, and advocates for better access to mental health practitioners. It is also necessary for researchers to examine the effects of innovative techniques on behavior and patient outcomes to generate larger government funds to shed light on mental health services in rural areas.
